# RNA-Seq reveals novel genes and pathways associated with hypoxia duration and tolerance in tomato root

**DOI:** 10.1038/s41598-020-57884-0

**Published:** 2020-02-03

**Authors:** Vajiheh Safavi-Rizi, Marco Herde, Christine Stöhr

**Affiliations:** 1 Department of Plant physiology, Institute of Botany and Landscape Ecology, University of Greifswald, Greifswald, Soldmannstrasse 15, D-17487 Greifswald, Germany; 2 Department of Molecular Nutrition and Biochemistry of Plants, Institute of Plant Nutrition, Leibniz University Hannover, Herrenhäuser Strasse 2, 30419 Hannover, Germany

**Keywords:** Plant sciences, Plant stress responses, Abiotic

## Abstract

Due to climate change, economically important crop plants will encounter flooding periods causing hypoxic stress more frequently. This may lead to reduced yields and endanger food security. As roots are the first organ to be affected by hypoxia, the ability to sense and respond to hypoxic stress is crucial. At the molecular level, therefore, fine-tuning the regulation of gene expression in the root is essential for hypoxia tolerance. Using an RNA-Seq approach, we investigated transcriptome modulation in tomato roots of the cultivar ‘Moneymaker’, in response to short- (6 h) and long-term (48 h) hypoxia. Hypoxia duration appeared to have a significant impact on gene expression such that the roots of five weeks old tomato plants showed a distinct time-dependent transcriptome response. We observed expression changes in 267 and 1421 genes under short- and long-term hypoxia, respectively. Among these, 243 genes experienced changed expression at both time points. We identified tomato genes with a potential role in aerenchyma formation which facilitates oxygen transport and may act as an escape mechanism enabling hypoxia tolerance. Moreover, we identified differentially regulated genes related to carbon and amino acid metabolism and redox homeostasis. Of particular interest were the differentially regulated transcription factors, which act as master regulators of downstream target genes involved in responses to short and/or long-term hypoxia. Our data suggest a temporal metabolic and anatomic adjustment to hypoxia in tomato root which requires further investigation. We propose that the regulated genes identified in this study are good candidates for further studies regarding hypoxia tolerance in tomato or other crops.

## Introduction

Global climate change has been associated with frequent flooding periods over the past 60 years. Flooding has negative consequences for crop yields, with reductions of up to 10% or 40% in severe cases causing a decrease in food supply for an increasing human population^1,2^. Therefore, the development of flood tolerant cultivars is crucial for sustainable agriculture. Although some improvements in flood tolerance have been achieved through natural genetic variation, e.g. *Submergence 1 (SUB1)* gene in rice, flood tolerance in other crops requires further investigation^3^.

One component of flood stress is oxygen deprivation, or hypoxia, which is due to the *ca*. 10^4^ fold lower diffusion rate of oxygen in water than in air^3^. Oxygen deficiency can also occur in tissues with high metabolic activity as well as bulky or dense tissues such as phloem, meristems, seeds and fruits^1^. Hypoxia is associated with disturbances in energy supply due to the inhibition of mitochondrial respiration, pH alteration, changes in redox state, reactive oxygen (ROS) accumulation and Ca^+2^ spiking^4,5^. A set of 49 hypoxia-induced genes encoding transcription factors (TF) as well as metabolic activities such as glycolysis and fermentation has been identified in *Arabidopsis thaliana*^6^. Moreover, it has been shown that hypoxia is important for general plant development such as shoot meristem activity and root architecture^7,8^.

Hypoxia sensing and signalling in *Arabidopsis thaliana* is conducted via the oxygen- and NO-dependent N-degron pathway which regulates the proteolysis or stability of subgroup VII ETHYLENE-RESPONSE FACTOR (ERFVII) TFs, such as HYPOXIA RESPONSIVE1 and 2 (HRE1 and HRE2) and RAP2.12. During the first step in this pathway, methionine (Met) aminopeptidase cleaves the N-terminal Met. The cysteine (Cys) will be oxidized by plant cysteine oxidase (PCO) and an arginyl (Arg)-tRNA transferase will add an Arg to the oxidized Cys. Eventually, the protein is targeted by PROTEOLYSIS 6 (PRT6) or other E3 ligases for ubiquitination and degradation by 26 S proteasome. Oxidation of Cys via PCO activity requires oxygen and therefore controls the stability of ERFVII TFs^3,9–12^.

The precise mechanism of the role played by NO in ERFVII degradation through the N-degron pathway is not yet unravelled. However, it is clear that MC-ERFVII TFs are degraded when NO is available via the N-degron pathway and are stabilized when NO is removed, either pharmaceutically or genetically^13^. Recently, it has been shown that prior to hypoxia, ethylene mediates NO scavenging via induction of PHYTOGLOBIN1 (PGB1) and therefore ERFVII stability. This process is important for pre-adaption of plants to the upcoming hypoxia^14^.

Comparative transcriptomics has been applied to the study of hypoxic stress responses in several plant species, including *Arabidopsis*, poplar, rice and soybean^15,16^. In a former study, the authors focused on species specific molecular and metabolic responses to hypoxia by comparing nine metabolome profiling studies as well as expression profiles of genes encoding enzymes involved in the metabolic pathways of rice, *Arabidopsis* and poplar^16^. They also studied full transcriptome profiles of the three above-mentioned species to compare the inter-specific expression pattern of orthologous genes. Interestingly, the most highly regulated genes found in any one of the species were either not responsive to hypoxia or did not have any orthologues in the other species. Overall, there was a divergent expression profile in response to hypoxia among the different species. Therefore, the authors concluded that the regulation of the metabolic response involved in hypoxia tolerance at the transcriptional level is species specific, especially with regard to signalling and post-transcriptional regulation. This finding indicates the importance of species-specific studies for the identification of genes involved in hypoxia tolerance.

Recently, an elaborate study was conducted to compare translatome and epigenome dynamics between transient and progressive hypoxia/reoxygenation conditions in plants^17^. The authors discovered that transient hypoxia resulted in the induction of some of the genes from the epigenome to the translatome while some of the other genes showed only epigenomic changes and required progressive hypoxia for translatome induction. This study indicates that multi-level dynamics between the nucleus and cytoplasm are required for distinctive responses to transient and prolonged hypoxia stress. Moreover, another recent in-depth study was conducted to investigate flooding induced plasticity in response to submergence among different species including rice, the domesticated tomato and it’s wild relative (*Solanum pennellii* (Sp)) which is adapted to dryland^18^. The authors studied transcriptional and post-transcriptional modulations in seedling root tips in response to short term (2 h) submergence. The data indicated that there is conversion not only in regulated genes but also cis regulation by four transcription factor families. However, there were still some species specific responses concerning chromatin accessibility and the level of submergence activation observed in wetland plants.

Due to its high lycopene content, with anti-oxidative properties, the tomato (*Solanum lycopersicum* L.) is one of the most nutritionally and economically important crop plants globally. Its production is affected by various abiotic stresses^19,20^. The tomato has been considered as one of the models for genetic studies in dicotyledonous crops^21^. Since the completion of its genome^22^, there has been a significant increase in the use of the tomato as a model for agricultural research^19^. The tomato is considered to be sensitive to flood stress^2^. Flood tolerant genotypes exhibit different physiological, anatomical and morphological properties, such as adventitious root and aerenchyma formation. In tomatoes, leaf chlorosis, epinasty, necrosis and reduced fruit yield are associated with the response to flood stress^2,23^. Apart from age, soil properties and other internal and external factors such as flood duration have been shown to strongly influence flood tolerance in different plant species and genotypes^19,24,25^. Although former studies have investigated the physiological and morphological response of different tomato genotypes to flooding and hypoxic stress^26,27^, the transcriptomic response of tomato roots to prolonged hypoxia has not yet been addressed. Moreover, most of the studies conducted on hypoxic stress adaption are on *Arabidopsis* and rice. Therefore, there is a knowledge gap regarding the underlying molecular mechanism involved in hypoxia tolerance in tomato.

The aim of the current study was to investigate the effects of short and prolonged hypoxia, an inevitable consequence of flood stress, on the regulation of gene expression in tomato root (*cv*. Moneymaker). Moneymaker is a non-hybrid and non commercial tomato cultivar which it’s genome sequence is available at Sol Genomics network. This more in-depth study enabled us to identify the molecular players involved in tomato root responses to short- and long-term hypoxia. These results have the potential to be further used by breeders and scientists for developing cultivars with improved hypoxia tolerance and increased yield production under flood stress.

## Results

### High throughput sequencing of root RNA samples

High throughput sequencing results of 12 samples, including hypoxia treatments and multiple controls are summarized in Table 1. Following the pre-processing steps of adapter clipping and low base quality filtering, ~254 and 250 million high quality reads remained in total (ca. 42 and 41 million reads per sample), for 6 h and 48 h treatments respectively. Of these, ca. 238 and 237 million reads for the two treatments, (93.5% and 94.7% for each sample), were mapped onto the tomato reference genome (ITAG2.4), using Genomics workbench V7.5.5 **(**Table 1**)**.

**Table 1:** Mapping statistics of RNA-Seq.

Sample Nr.	Treatment	Sample ID	Total reads	Mapped reads	Mapping rate (%)	Unique match	Multi-position match
1	Control_6 h	CR-A-6_R1_clipped	56403530	53306446	94.51	17585641	35720805
2	Control_6 h	CR-B-6_R1_clipped	52762601	49993112	94.75	16181942	33811170
3	Control_6 h	CR-C-6_R1_clipped	28244134	26253009	92.95	8512535	17740474
4	Hypoxia_6 h	HR-A-6_R1_clipped	15791167	14504306	91.85	4915794	9588512
5	Hypoxia_6 h	HR-B-6_R1_clipped	39867562	37302068	93.56	12158291	25143777
6	Hypoxia_6 h	HR-C-6_R1_clipped	61371105	57402023	93.53	18321583	39080440
7	Control_48 h	CR-A-48_R1_clipped	36325504	34452388	94.84	11271017	23181371
8	Control_48 h	CR-B-48_R1_clipped	32284452	30565595	94.68	9921005	20644590
9	Control_48 h	CR-C-48_R1_clipped	47173117	45065028	95.53	14666684	30398344
10	Hypoxia_48 h	HR-A-48_R1_clipped	48832418	46249059	94.71	15319583	30929476
11	Hypoxia_48 h	HR-B-48_R1_clipped	40650352	38583977	94.92	13102447	25481530
12	Hypoxia_48 h	HR-C-48_R1_clipped	45662549	42904687	93.96	13993004	28911683
**Total_6 h**			254440099	238760964		77675786	161085178
**Average_6 h**			42406683.2	39793494	93.53	12945964.33	26847529.67
**Total_48 h**			250928392	237820734		78273740	159546994
**Average_48 h**			41821398.7	39636789	94.77	13045623.33	26591165.67

### Short- and long-term hypoxia elicited distinct responses in physiological parameters as well as in gene expression modulation

The aim of this study was to investigate the transcriptome modulation in tomato roots in response to short- and long-term hypoxic stress. Hypoxic stress was applied using nitrogen (N_2_) gas to the roots of five weeks old plants in a hydroponic system. The oxygen level before N_2_ treatment was 262 μM. After 80 min of N_2_ treatment, oxygen concentration decreased to 6.2 μM and remained stable. Based on aquatic definition of hypoxia, oxygen concentration below 156 μM.is defined as hypoxic^28^. Root samples were harvested after 6 h and 48 h after initiation of N_2_ treatment.

**Figure 1:**
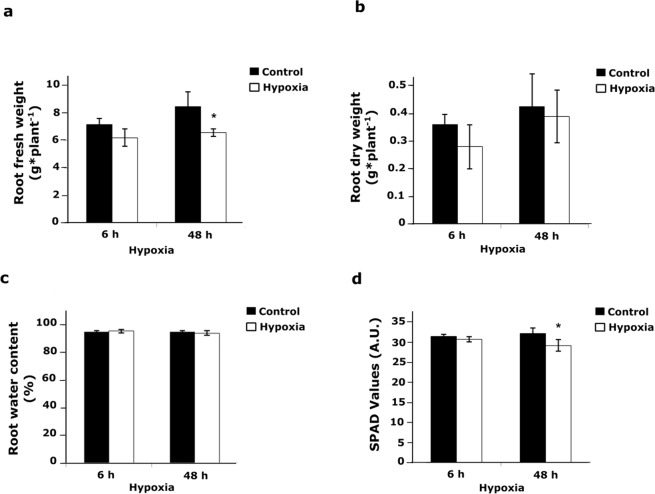
Fresh weight, dry weight and relative chlorophyll content of tomato plants under short- and long-term hypoxia compared to controls. (**a)** Fresh weight; (**b)** dry weight and **c** water content (%) of 5 week old tomato roots after 6 h and 48 h hypoxia, compared to controls. (**d)** Relative chlorophyll content in leaf #3 of plants under hypoxia conditions and controls are shown as SPAD values. Data represents means ± SD; n = 3; *, Significant differences (Student’s t-test, P < 0.05).

No significant differences were observed between short term hypoxia treatments and controls in fresh and dry weight differences of roots **(**Fig. 1a,b**)**, water content (%) and SPAD values **(**Fig. 1c,d**)**. As anticipated, these results indicate that 6 h hypoxia is not long enough to affect the above-mentioned parameters. However, hypoxia resulted in a significantly lower root fresh weight after 48 h **(**Fig. 1a**)**. Nonetheless, no statistically significant differences were observed in response to hypoxia (48 h) for root dry weight and root water content (%) **(**Fig. 1b,c**)**. Moreover, after 48 h, relative chlorophyll levels, represented by SPAD values, showed a significant (P < 0.05) reduction in response to hypoxia (Fig. 1d). This indicated that long-term hypoxia at the root could have an impact on leaf performance.

**Figure 2:**
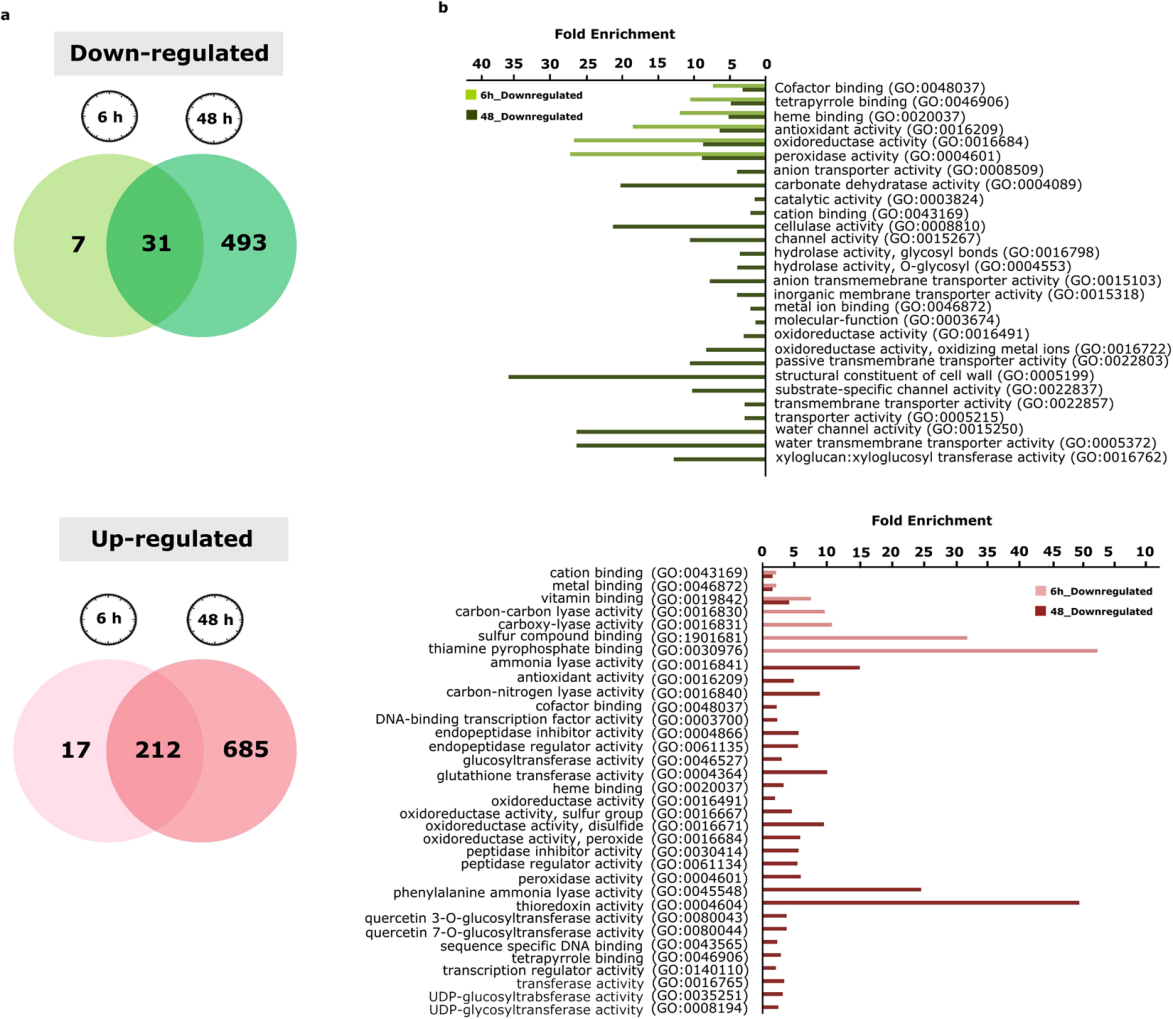
Transcriptomic modulation in tomato roots in response to 6 h and 48 h hypoxia. (**a)** Venn diagrams display the number of up-regulated (light and dark red) and down-regulated (light and dark green) genes. Light and dark colours represent 6 and 48 h hypoxia, respectively. Only genes with significant ( ≥ 2-fold) expression changes and Padj < 0.05 are depicted. (**b**) Enriched GO terms (Padj < 0.05), describing molecular function, among down- and up-regulated genes in response to 6 h and 48 h hypoxia. The regulated genes in all samples were analysed for enriched GO terms using the online tool PANTHER 14.0 and *Solanum lycopersicum* as a reference organism. The light and dark green bars represent all significantly enriched GO terms associated with down-regulated genes in response to 6 h and 48 h hypoxia, respectively. The light and dark red bars represent all significantly enriched GO terms associated with up-regulated genes in 6 h and 48 h hypoxia samples, respectively.

We observed that short-term hypoxia (6 h) resulted in regulation changes in only 267 genes (38 down-regulated and 229 up-regulated) while long-term hypoxia (48 h) resulted in regulation changes of 1421 genes (524 down-regulated and 897 up-regulated) **(**Fig. 2a**)**. The fact that long-term hypoxia led to a fivefold higher number of regulated genes indicated that hypoxic stress duration has a strong impact on adaptive responses induced by gene expression.

### Functional classification of differentially regulated genes using GO terms and MapMan categories

To identify the most highly regulated hypoxia-responsive pathways in response to short- and long-term stress, a Gene Ontology (GO) term enrichment analysis (molecular function, biological process and cellular compartment) was performed on the up- and down-regulated genes. To visualize substantially regulated GO terms after 6 h and 48 h hypoxia, all significantly enriched (Padj < 0.05) GO terms describing molecular function for the up- and down-regulated genes are displayed **(**Fig. 2b**)**. The list of all the GO terms associated with the regulated genes is provided in Supplementary Table 1. It must be noted that GO terms refer to the proteins encoded by the genes and therefore in some cases, the word “activity” is used in GO term results.

GO term analysis revealed that down-regulated genes encoded proteins with peroxidase and oxidoreductase activity, at both time points. Among those GO terms, which were associated only with down-regulated genes at 48 h, we observed that cell wall, water channel and transmembrane transporter activity showed the highest enrichment. Moreover, the up-regulated genes encoded for proteins that were involved in cation binding, metal ion binding and vitamin binding at both time points. Thiamine pyrophosphate binding and sulfur compound binding showed the highest enrichment at 6 h only while thioredoxin activity and phenylalanine ammonia-lyase activity represented the maximum enrichment in up-regulated genes at 48 h. It was noteworthy that genes up-regulated at 48 h only and not 6 h, were enriched in oxidoreductase activity and transcription factor activity, although these did not represent the highest enrichment levels found.

These results indicate that the short- and long-term hypoxia had distinct and also conserved effects on cellular activities associated with different stress response pathways such as cell wall, redox homeostasis and water transport. Moreover, regulation of transcription appears to be more active at later time points (>6 h hypoxia) resulting in modulation of hypoxia responsive genes.

MapMan categories^29^ based on ITAG2.3 annotations, were used to provide independent verification of results and to gain a more in-depth view of the biological pathways related to differentially regulated genes **(**Supplementary Table 2**)**. We compared the result of both GO terms **(**Supplementary Table 1**)** and MapMan categories **(**Supplementary Table 2**)** related to regulated genes in our study. We observed similarities between GO terms and MapMan categories related to cell wall, antioxidant activity, oxidoreductase activity, organic acid metabolic process, glycolytic process (glycolysis) and reactive oxygen species metabolic processes. However, only MapMan categories were used for the description of the regulated genes in our study.

### Validation of differentially expressed genes in response to hypoxia using qPCR

**Figure 3:**
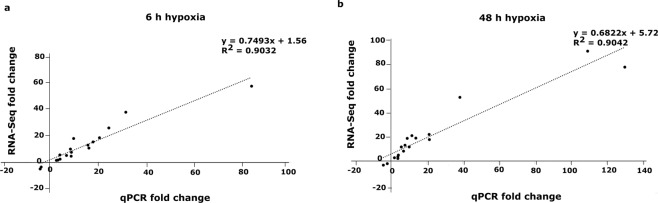
Validation of RNA-Seq data using qPCR. Strong positive correlation of 18 differentially regulated genes (expression ratios ≥2 and Padj <0.05, n = 3) between RNA-Seq and qPCR data at (**a**) 6 h (R^2^ = 0.90) and (**b**) 48 h (R^2^ = 0.90) after hypoxia. Fold-changes represent the expression changes of each gene under hypoxia (6- or 48 h) relative to its respective control, (n = 3).

Validation of RNA-Seq data was performed using qPCR on 18 hypoxia responsive genes at both 6 h and 48 h, excluding two genes, which were up-regulated only at 48 h (*PGB1* and *PGB3*) **(**Supplementary Table 4**)**. We observed a positive correlation between fold-change values (hypoxia/control) of RNA-Seq and qPCR data for both 6 h (r^2^ = 0.90) and 48 h (r^2^ = 0.90) time points **(**Fig. 3a,b**)**. These data confirm the reliability of the RNA-Seq results used in this study. The expression fold-changes of the 18 selected genes for RNA-Seq and qPCR are provided in Supplementary Table 3.

### Short- and long-term hypoxia regulates expression of genes involved in carbon (C) flux and amino acid metabolism

**Figure 4:**
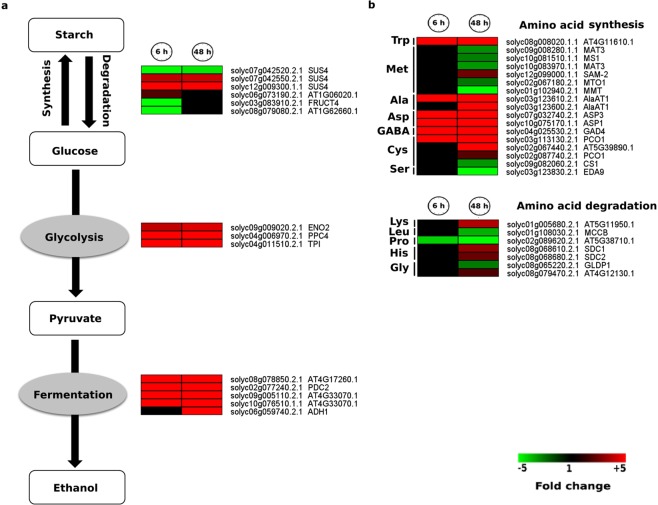
Hypoxia-induced and repressed carbon flux and amino acid metabolism associated genes. Heat maps display the up-regulated (red bars) or down-regulated (green bars) tomato genes. (**a)** glycolysis and fermentation; (**b**) amino acid metabolism in response to 6 h and 48 h hypoxia and their Arabidopsis thaliana homologs. Genes with expression ratios ≥2 and Padj <0.05 (n = 3) are depicted.

We observed that three tomato genes annotated as *SUS4* showed regulation changes (one down-regulated and two up-regulated) at 6 and 48 h hypoxia **(**Fig. 4a**)**. Moreover, several genes coding for enzymes, which are involved in glycolysis (*ENO2*, *PPC4* and *TPI*) and fermentation (*PDC2*, *ADH1*), were up-regulated at both time points in response to hypoxic stress **(**Fig. 4a**)**. These data indicate that induction of the genes encoding enzymes involved in alternative routes for ATP production is one of the earliest responses under hypoxia.

Alanine (Ala) and 2-oxoglutarate (2OG) as well as gamma-aminobutyric acid (GABA) shunts are amino acid related pathways leading to accumulation of Ala, GABA and succinate upon hypoxia^30^. Among different regulated genes involved in amino acid synthesis and degradation **(**Fig. 4b**)**, two transcripts encoding AlaAT showed up-regulation in response to hypoxia. However, one transcript (solyc0g123610.2.1) was up-regulated only at 6 h hypoxia while the other one (solyc0g123600.2.1) showed up-regulation at both time points. Furthermore, a transcript encoding glutamate decarboxylase 4 (*GAD4*) was up-regulated at 6 and 48 h hypoxia in our study. Further investigations are required to confirm the role of Ala- and GABA shunts in the acclimation of tomato roots to hypoxia.

### Hypoxia affects the link between N metabolism and NO formation/scavenging

NO formation under normoxic conditions is very limited due to the high ratio of NO_3_^−^ to NO_2_^−^ (50 to 100 fold) and NR preference for NO_3_^−^ rather than NO_2_^−^. However, hypoxia results in a higher accumulation of NO_2_^−^ and therefore a higher level of NO can be produced via the NR pathway and mitochondrial cytochrome c oxidase^31–33^.

**Figure 5:**
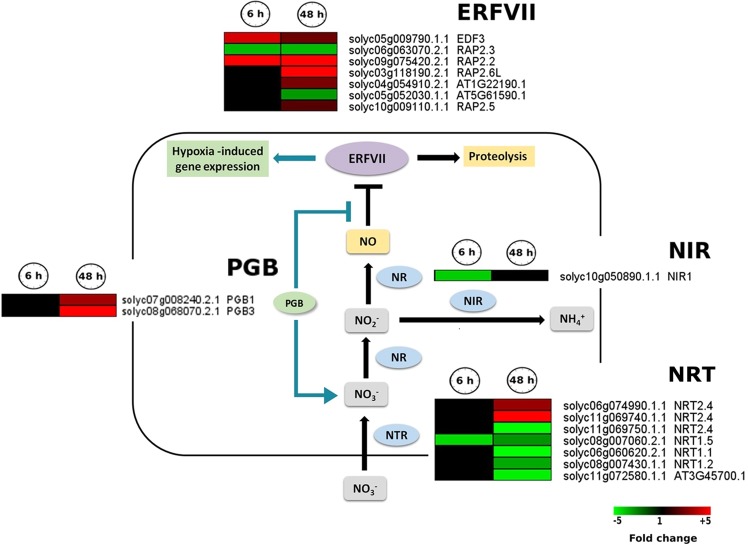
Transcriptional changes of genes related to NO production and primary nitrogen metabolism. Heat map of regulated genes involved in NO production (*NR*) and scavenging (*PGBs*) as well as early steps of primary nitrogen metabolism (*NR*, *NIR* and *NRTs*). The heat maps display the up-regulated (red bars) or down-regulated (green bars) tomato genes in response to 6 h and 48 h hypoxia and their Arabidopsis thaliana homologs. Genes with expression ratios ≥2 and Padj <0.05 (n = 3) are depicted. NO, nitric oxide; PGB, phytoglobin; NR, nitrate reductase; NIR, nitrite reductase; NRT, nitrate transporter.

We investigated the effect of hypoxia on N metabolism related processes such as induction of genes encoding NO_3_^−^ transporters, nitrate and nitrite reductase as well as nitric oxide (NO) scavenging via phytoglobin (PGB). We observed that a transcript encoding NO_2_^−^ reductase 1 (*NIR1*) was down-regulated after 6 h hypoxia, leading to accumulation of NO_2_^−^ **(**Fig. 5**)**. It has been suggested that NO and its interaction with PGB might play a role in hypoxic stress adaption^34^. We observed that two PGB encoding transcripts, *PGB1* and *PGB3*, showed up-regulation (3.5 fold and 5 fold, respectively) after 48 h hypoxia (Fig. 5).

Seven high- and low affinity NO_3_^−^ transporter encoding (NRTs) transcripts showed expression changes, mostly after 48 hypoxia. The only exception was *NRT1.5*, which was down-regulated at 6 h and 48 h post-hypoxia. Out of three transcripts annotated as *NRT2.4*, two were up-regulated. The other *NRT2.4* as well as *NRT1.1*, *NRT1.2* and an uncharacterized NO_3_^−^ transporter gene were down-regulated after 48 h hypoxia **(**Fig. 5**)**.

**Figure 6:**
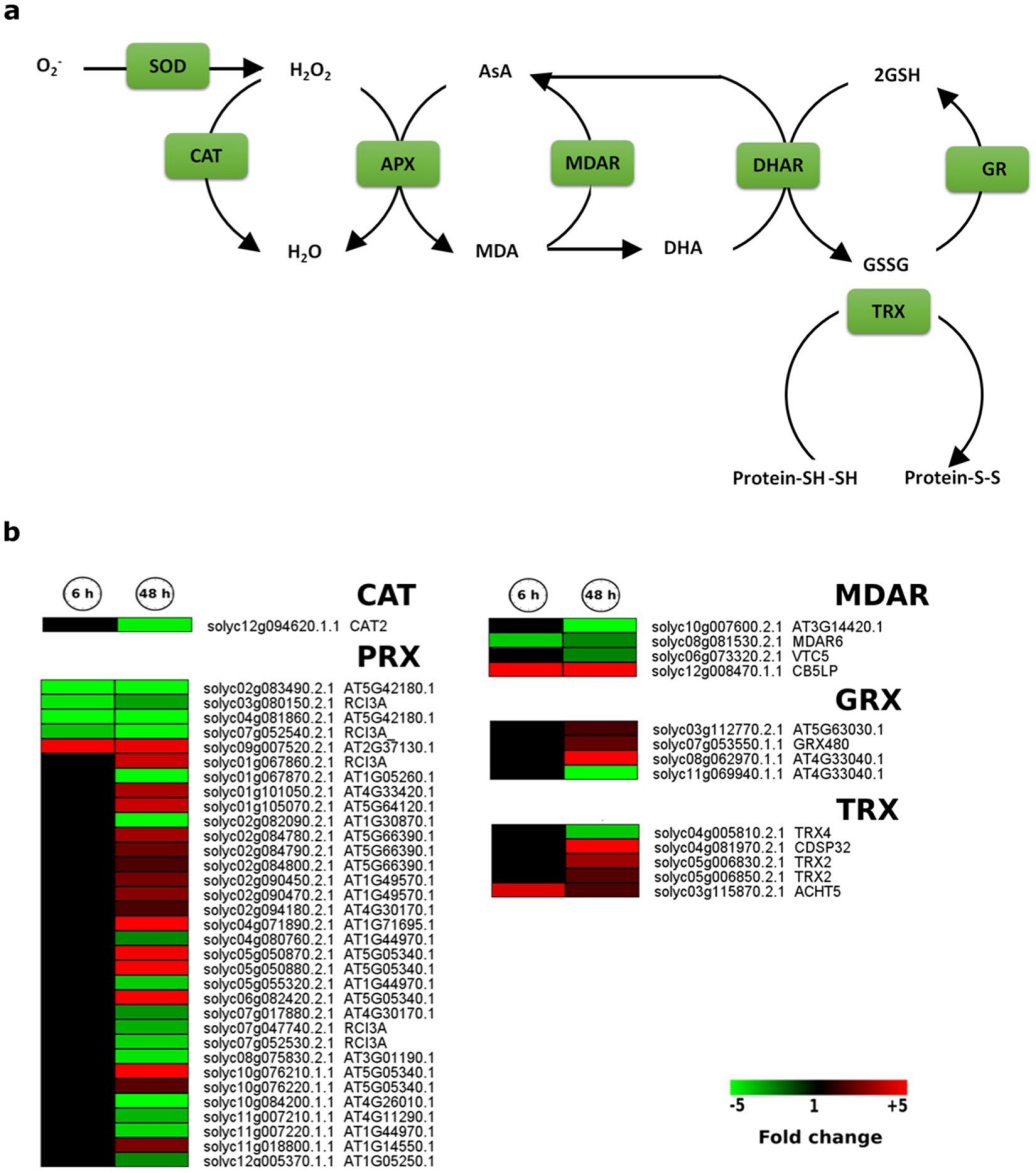
Expression pattern of genes encoding members of different antioxidant classes. (**a)** Ascorbate-glutathion cycle, modified from^86^. (**b)** Heat maps represent the up-regulated (red bars) or down-regulated (green bars) tomato genes in response to 6 h and 48 h hypoxia and their Arabidopsis thaliana homologs. Genes with expression ratios ≥2 and Padj <0.05 (n = 3) are depicted.

Altogether, these results suggest that N metabolism might be involved in NO production and scavenging in tomato root adaption to hypoxic stress with a gene regulation specific response to the duration of hypoxia **(**Figs. 5 and 6**)**. However, a precise measurement of NO level and N metabolites, in particular NO_2_^−^ under hypoxia is necessary to confirm their role in hypoxia responses in tomato roots.

### Redox related gene expression changes appear to be responsive to long-term hypoxic stress

**Figure 7:**
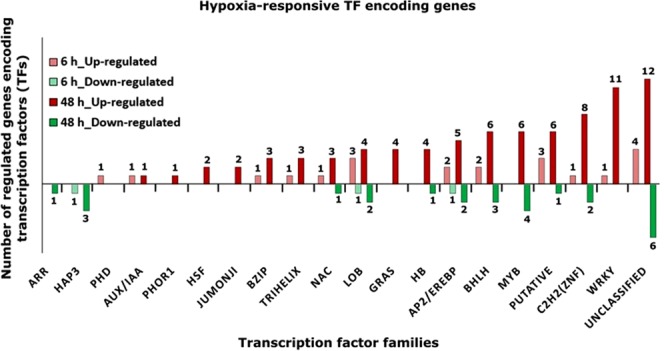
Hypoxia responsive TF encoding genes. Number of differentially regulated genes belonging to different TF families in response to 6 h and 48 h hypoxia. Genes with expression ratios ≥2 and Padj <0.05 (n = 3) are depicted.

ROS production is associated with low oxygen signalling and adaption in plants^35^. In the current study, genes encoding members of several redox-related enzyme families showed time-dependent regulation changes in response to hypoxia: catalase (CAT, one gene), peroxidase (PRX, 33 genes), monodehydroascorbate reductase (MDAR, 4 genes), glutathione reductase (GRX, 4 genes), thioredoxin (TRX, 5 genes). Eight genes encoding members of PRX, MDAR and TRX, were regulated at 6 h (3 up-regulated and 5 down-regulated). It was noteworthy that 47 redox-related genes were regulated after 48 h hypoxia with 22 and 25 genes being down- and up-regulated, respectively **(**Fig. 7**)**. Moreover, it was observed that out of 5 regulated TRX transcripts, 4 showed up-regulation **(**Fig. 7**)**.

Overall, our results indicate that there was a fine-tuning of redox homeostasis, which was dependent upon the duration of hypoxic stress.

### Transcription factors as key regulators of the response to short- and long-term hypoxic stress

**Figure 8:**
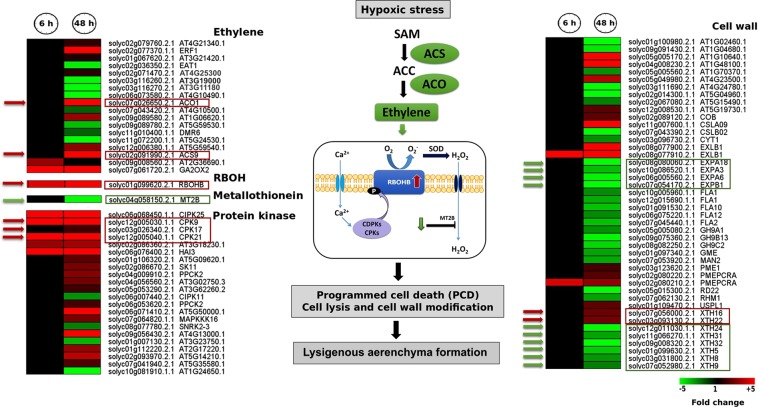
A proposed model of the biological processes and genes potentially involved in hypoxia inducible aerenchyma formation in tomato roots under short- and long-term hypoxia, modified from former studies on rice^62^. Low oxygen conditions result in the induction of genes encoding ethylene biosynthesis enzymes *ACS9* and *ACO1*. Moreover, expression of *RBOHB*, which is involved in the production of O_2_^−^ radicals from oxygen in the apoplast; increases under low oxygen. On the other hand, hypoxia may stimulate Ca^2+^ influx from the apoplast to the cytosol resulting in direct stimulation of RBOHB via its EF-hand motif and higher activity of group I CDPK encoding genes such as *CPK9*, *CPK7* and *CPK21*. RBOHB phosphorylation may enhance its activity and may increase the level of ROS in the apoplast as well as cytosol. Moreover, the expression of the genes encoding ROS scavengers such as *MT2B* decreases, potentially leading to ROS accumulation and PCD induction for lysigenous aerenchyma formation in root cortical cells. Heat maps display the up-regulated (red bars) or down-regulated (green bars) tomato genes in response to 6 h and 48 h hypoxia and their *Arabidopsis thaliana* homologs. Genes with expression ratios ≥2 and Padj <0.05 (n = 3) are depicted. Red and green boxes with arrows refer to the formerly reported categories of aerenchyma formation genes related to ethylene biosynthesis, RBOH, metallothionein, protein kinase and cell wall, based on^62,63,87–89^. SAM, S-adenosyl-l-methionine; ACS, 1-aminocyclopropane-1-carboxylic acid (ACC) synthase; ACC, 1-aminocyclopropane-1-carboxylic acid; ACO, and ACC oxidase; SOD, Superoxide dismutase; RBOH, respiratory burst oxidase homolog (RBOH) isoform B; O_2_^−^, superoxide anion; Ca^2+^, Calcium; CDPK, Ca^2+^-dependent protein kinases; MT2B, Metallothionein-like protein 2B; ROS, reactive oxygen species; XTH, xyloglucan endotransglucosylase/hydrolase.

Ethylene response factor (ERF) TFs, in particular ERFVII, belonging to the APETALA2/ethylene response factor (AP2/EREBP), are among the most studied TFs known to be involved in hypoxic response. However, there is evidence that the huge transcriptome modulation observed under low oxygen is not limited to ERF TF targets^36^. Therefore, we investigated gene expression changes of members of different transcription factor families in response to short- and long-term hypoxic stress. A total of 122 tomato transcripts annotated as TF belonging to different families showed regulation changes at 6 h (25 genes, 23 up- and 2 down-regulated) and 48 h (118 genes, 89 up- and 29 down-regulated), with 21 TF encoding genes being regulated at both time points **(**Fig. 8 and Supplementary Table 3**)**.

Seven genes belonging to the AP2/EREBP family were regulated in our study; three genes (*EDF3*, *RAP2.2* and *RAP2.3*) at both 6 h and 48 h and 4 genes (*RAP2.6* *L*, Solyc04g054910.2, Solyc052030.1 and *RAP2.5*) only at 48 h after hypoxic stress. Moreover, members of different TF families such as *Arabidopsis* response regulators, ARR (1 gene), PHOR (1 gene), Heat shock transcription factor, HSF (2 genes), JUMONJI (2 genes), GRAS (4 genes), homeobox, HB (5 genes) and MYB (9 genes) were responsive only to long-term (48 h) hypoxic stress. Members of WRKY (1, 11 genes), NAC (1, 4 genes), basic helix loop helix, bHLH (2, 8 genes), C2H2-zinc finger (1, 10 genes) and bZIP (1, 3 genes) families were responsive to both short and long-term hypoxia.

These data indicate a fine-tune regulation of TFs which was sensitive to the duration of hypoxic stress. The hypoxic responsive TF encoding genes are particularly interesting candidates for hypoxia tolerance studies due to their crucial role as master regulators of stress response.

### Hypoxia resulted in regulation of the genes linked to ethylene biosynthesis, ROS production and PCD

Aerenchyma formation through programmed cell death (PCD) in root cortical cells is a coping strategy under hypoxic conditions. PCD is the consequence of ethylene accumulation and up-regulation of *respiratory burst oxidase* (*RBOH*) genes involved in ROS production^37^.

We investigated the expression changes of rice orthologue genes in tomato root with potential roles in aerenchyma formation.

**Figure 9:**
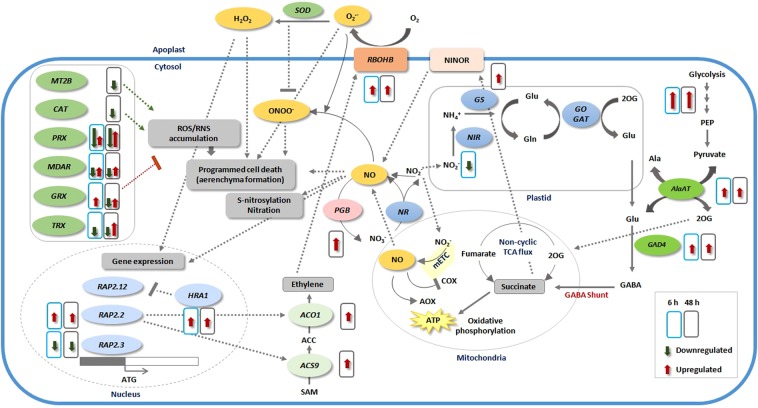
Schematic model illustrating the main transcriptome modulations in tomato root in response to short- and long-term hypoxia. Only enzymes involved in the main pathways are shown. Solid line arrows indicate enzymatic reactions; dashed line arrows indicate induction of a certain process, while flat-headed dashed lines represent inhibition of a certain process. When more than one gene is involved in a category, the length of red and green arrows is in proportion to the number of up- and down-regulated genes, respectively. NR, nitrate reductase; NIR, nitrite reductase; GS, glutamine synthetase; GOGAT, glutamine oxoglutarate aminotransferase; AlaAT, alanine aminotransferase; GAD, glutamate decarboxylase; PEP, Phosphoenolpyruvate; GABA, gamma-Aminobutyric acid; 2OG, 2-oxoglutarate; COX, cytochrome c oxidase; mETC, mitochondrial electron transport chain; AOX, alternative oxidase; ATP, adenosine triphosphate; NO, nitric oxide; PGB, phytoglobin; RBOHB, respiratory burst oxidase homolog protein B; NINOR, nitrite:NO reductase; SOD, superoxide dismutase; ACO, aminocyclopropane carboxylate oxidase; ACS, Acetyl-coenzyme A synthetase; MT2B, metallothionein-like protein 2B; CAT, catalase; PRX, peroxiredoxin; MDAR, monodehydroascorbate reductase; GRX, Glutaredoxin; TRX,Thioredoxin reductases; HRA1, hypoxia response attenuator 1; O_2_, oxygen; O_2_^−^, superoxide anion; H_2_O_2_, hydrogen peroxidase; ONOO^−^, Peroxynitrite; RAP, related to apetala; Gln, glutamine; Glu, glutamate; Ala, alanine; NO_2_^−^, nitrite; NO_3_^−^, nitrate; NH_4_^+^, ammonium.

Our data analysis showed that genes encoding ethylene biosynthesis enzymes underwent upregulation in response to 48 h hypoxia e.g. *ACS*9 (>13 fold) and *ACO1* (ca. 45 fold). We observed that out of eight members of the *RBOH* gene family in tomato (*RBOHA* to *RBOHH*), *RBOHB* was strongly up-regulated (ca. 18 and 53 fold after 6 h and 48 h hypoxia, respectively) **(**Fig. 9). A methallothionein encoding gene (*MT2B*, encoding metallothionein-like protein 2B), involved in ROS scavenging, was down-regulated after 48 h of hypoxia **(**Fig. 9). *MT2B* down-regulation might lead to ROS accumulation and subsequent aerenchyma formation, as it has been reported in rice^38^.

Among diverse regulated protein kinase encoding genes, we observed up-regulation of several CPKs transcripts: *CPK*9*, CPK17 and CPK21*. This up-regulation was observed at 6 h as well as 48 h after hypoxia **(**Fig. 9**)**.

Among regulated transcripts encoding cell wall proteins, we observed 4 EXP encoding genes (*EXPA18*, *EXPA3*, *EXPA6* and *EXPB1*) to be down-regulated after 48 h hypoxia. In total, eight XTH encoding genes showed expression changes in the current study with six (*XTH24*, *XTH31*, *XTH32*, *XTH5*, *XTH8* and *XTH9*) being down-regulated and two (*XTH16* and *XTH22*) being up-regulated **(**Fig. 9**)**. Among cell wall related up-regulated genes, an expansin-like encoding gene, *EXLB1*, showed the highest up-regulation at 6 h (>24 fold) with a slight decrease (ca. 12 fold) at 48 h. Moreover, the cellulose-synthase-like *CSLA09*, was another highly up-regulated gene (>20 fold) at 48 h.

## Discussion

### Transcriptome reprogramming in response to short- and long-term hypoxic stress

The root is the first organ to experience hypoxic stress under flood conditions. Hypoxia, due to lowered oxygen transport under flood stress, is particularly damaging to the root because of the lack of photosynthetic oxygen production^39^. The tomato is a widely used crop plant. However, little is known about the underlying molecular mechanism associated with hypoxia tolerance in tomato roots.

The current study utilises comparative transcriptomics to elucidate the differential gene expression taking place in tomato (cv. Money maker) roots in response to short- (6 h) and long-term (48 h) hypoxia. Long-term hypoxia resulted in significantly (P<0.05) lower fresh weight and chlorophyll content (SPAD values), (Fig. 1a,d**)** while short-term hypoxia did not show any significant effect on the above-mentioned parameters.

Overall, we observed a ~50 times higher number of regulated genes at 48 h compared to 6 h hypoxia **(**Fig. 2a**)**. This substantial discrepancy indicates that a major shift occurs in transcriptome response after2 days of hypoxia, highlighting the importance of hypoxic stress duration. It was also apparent that the number of up-regulated genes in response to hypoxia was higher than down-regulated genes at both time points.

To validate the RNA-Seq data, we confirmed the expression changes in 18 differentially regulated genes using qPCR. We observed a high level of consistency between the qPCR and RNA-Seq expression data (Fig. 3a,b) showing a strong positive correlation at both time points (r^2^ = 0.90), indicating the reliability of our transcriptome data.

### C flux and amino acid metabolism reconfiguration in response to hypoxic stress

The regulation of gene expression involved in primary metabolism and energy homeostasis is crucial to avoid the detrimental effects of mitochondrial oxidative phosphorylation inhibition under hypoxic stress^40^. The core hypoxia responsive genes related to primary carbon metabolism reconfiguration such as *alcohol dehydrogenase* (*ALDH)*, *pyruvate decarboxylase* (*PDC2*) and *sucrose synthase* (*SUS4*) showed up-regulation in our study (Fig. 4a). Induction of the above-mentioned gene expression in different plant species under hypoxic stress has been reported. Additionally, it has been reported that *ADH*, *PDC* as well as *SUS* mutant plants exhibit reduced hypoxia tolerance^6,40,41^.

**Figure 10:**
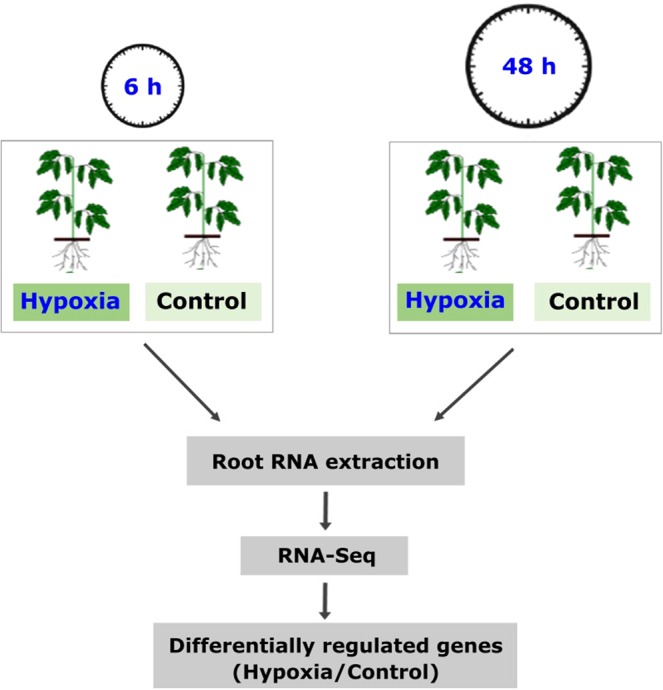
Experimental set-up. A schematic representation of the experimental set-up is provided. Roots of five weeks old tomato (*Solanum lycopersicum*, cv. Moneymaker) plants were exposed to hypoxia for 6 h or 48 h. N_2_ gas was applied to induce hypoxia in a hydroponic system. RNA was extracted from the roots of control and hypoxia treated plants for RNA-Seq analysis.

Aside from carbon metabolism related gene expression changes, we observed that genes encoding enzymes with pivotal roles in N metabolism showed up-regulation in response to hypoxia, e.g. *alanine aminotransferase* (*AlaAT1*, two transcripts), a key enzyme of amino acid metabolism and *GAD4*, encoding another key enzyme involved in GABA shunt, (Figs. 4b and 10). *GAD4* and one of the two transcripts annotated as *AlaAT* were up-regulated at both time points while the other AlaAT encoding transcript showed up-regulation only at 48 h.

The AlaAT enzyme reversibly transfers an amino group from glutamate to pyruvate leading to the formation of 2-oxoglutarate and Ala^40,42^. Ala accumulation and up-regulation of the *AlaAT* gene has been observed in different plant species under hypoxic stress^43,44^. However, despite proline and betaine, Ala may not function as a protectant. It has been proposed that under hypoxia, the AlaAT/NADH-GOGAT cycle uses glycolysis produced pyruvate to store carbon in the form of Ala synthesized by AlaAT. In this process, glutamate provides the amino group while 2-oxoglutarate is consumed by NADH-GOGAT leading to glutamate and NAD^+^ regeneration. Ala accumulation under hypoxic conditions will be beneficial for post-hypoxic conditions by providing pyruvate and glutamate via the AlaAT reverse reaction^40^.

The finding of up-regulation of glutamate decarboxylase (*GAD4*) in our study, one of the key enzymes in the GABA shunt, is in line with former studies in *Arabidopsis thaliana* which showed among the five homologous *GAD*, only *GAD4* is induced upon hypoxia^43^. GABA production and its subsequent transamination to succinic semialdehyde might be beneficial for cytosolic pH stabilization as well as providing a route for conversion of pyruvate to Ala^3^. However, whether Ala and GABA accumulate under hypoxia and if they are involved in tomato root responses to hypoxia needs further investigation.

### Hypoxia-induced ROS production, redox associated gene expression and ethylene related genes

Oxidative stress response and redox signalling, including production of ROS and NO as well as other reactive nitrogen species (RNS), is associated with low oxygen stress^45,46^. Production of ROS signalling is associated with the enhanced activity of PM bound NADPH oxidases (*RBOH* gene), through Ca^+2^ signalling and phosphorylation, in response to stress perception^47,48^.

Apart from GRX and TRX related genes which showed more up-regulation, down-regulation was more frequent among the members of antioxidant related families such as CAT, PRX and MDAR at both time points in response to hypoxia, i.e.3 out of 4 and 4 out of 5 regulated genes, respectively (Fig. 7b**)**.

TRXs have been shown to be involved in several processes associated with oxidative proteins such as DNA damage related proteins, inducing activity in antioxidant protecting enzymes as well as in the regulation of scavenging mechanisms or signalling pathways in the antioxidant network. Moreover, TRXs have been shown to be involved in cold tolerance in rice^49^. However, the precise role of TRX members in response to hypoxia needs further investigation.

The expression of antioxidant genes (*SOD1*, *AOX1A*, *APX* and *MnSOD*) has been shown to play a role in aerenchyma development in wheat. It was shown that after 2 h hypoxia, antioxidant gene expression was up-regulated but down-regulated after 24 h^50^.

Among cell wall related up-regulated genes, we observed several members of the EXP and XTH families (Fig. 9). Different classes of cell wall proteins such as xyloglucan endotrans glucosylase (XET) and expansin (EXP) are involved in cell wall loosening at lower pHs, e.g. upon hypoxia during waterlogging. However, many cell wall related genes, among them XTH and EXP encoding genes, showed down-regulation, particularly at 48 h hypoxia (Fig. 9). This indicates that hypoxia did not strongly induce genes involved in cell wall degradation in our study.

NO production during hypoxia increases through different pathways including coordinated activity of the root-specific plasma membrane-bound nitrate reductase (PM-NR) and nitrite:NO reductase (NI-NOR)^51^, nitrate reductase (NR) and the mitochondrial electron transport chain^31,52^. The induction of NO after hypoxia in the root occurs rapidly, within minutes, and therefore it has been suggested to be involved in hypoxic stress response in the root^53^. Some of the functions, which have been attributed to hypoxia-induced NO, include a role in the production of ATP^54^, interaction with alternative oxidase (AOX)^55,56^ and programmed cell death (PCD) during aerenchyma formation^31,57^. Wany *et al*. (2017) have shown that NO, in addition to ROS, is involved in programmed cell death during ethylene-induced lysigenous aerenchyma formation under hypoxic stress in wheat roots.

We observed up-regulation of two PGB encoding transcripts, *PGB1* and *PGB3*, after 48 h hypoxia **(**Figs. 5 and 10). Up-regulation of both PGB encoding genes only after 48 h hypoxia might indicate that *PGB* expression in tomato roots is more a consequence of ATP level rather than as a direct response to O_2_ level, as has been suggested in former studies using respiratory chain- and ATP production inhibitors^58^. Non-symbiotic hemoglobins are induced in root exposed to hypoxic stress and are involved in NO scavenging leading to the formation of nitrosyl-phytoglobins after interaction with NO^58,59^. Moreover, it has been shown that overexpression of *PGB1* in *A. thaliana* enhances hypoxia tolerance and post-hypoxia survival^60^.

Apart from cell death-associated aerenchyma formation in cortex cells, the emergence of adventitious roots in rice is associated with epidermal cell death induced by ethylene as well as H_2_O_2_ and O_2_^•−^ which act as signalling molecules in this process^61^. In contrast with findings in *Arabidopsis* and rice, ethylene is not involved in early flood recognition and adaption to hypoxia in the tomato cultivar Moneymaker^14^. Concordantly, we observed that only long term hypoxia led to the induction of genes encoding ethylene biosynthesis enzymes *ACS9* and *ACO1* **(**Fig. 9). However, further studies are required to confirm ethylene accumulation in tomato roots under long term hypoxia. It has been reported that in rice roots, induction of *ACS1* and *ACO5* genes upon hypoxia results in ethylene biosynthesis, which in turn increases expression of *RBOHH*. Moreover, Ca^2+^ release from apoplast to cytosol results in RBOHH protein activation via protein kinases (CDPKs) such as CDPK5 and/or CDPK13. Conversion of apoplastic O_2_^•−^ to H_2_O_2_, whether enzymatically via superoxide dismutase (SOD) or non-enzymatically, enhances H_2_O_2_ levels and its transport to the cytosol. Concomitantly, down-regulation of ROS scavenger genes, such as type 1-Metallothionein (MT1) encoding gene, enhances ROS accumulation. Accumulation of ROS in the form of O_2_^•−^ and/or H_2_O_2_ leads to PCD and the formation of lysigenous aerenchyma in the cortical cells of rice roots^38,62,63^. In the current study, the *MT2B* encoding transcript, involved in ROS scavenging, showed down-regulation (>3 fold) after 48 h hypoxia. Former studies showed that constitutive down-regulation of *MT2B* transcript levels resulted in the accumulation of H_2_O_2_, indicating the potential role of MT2B in ROS scavenging. Moreover, an *MT2B* mutation has been shown to result in H_2_O_2_ accumulation and cell death associated with aerenchyma formation and adventitious root emergence in rice^38^.

### Regulated transcription factors serve as potential targets for improved hypoxia tolerance

Several TF families such as AP2/EREBP, MYB, WRKY, NAC and bZIP have been shown to be associated with different abiotic stresses^64^. Members of these families showed regulation changes in response to hypoxia in our study **(**Fig. 8 and Supplementary Table 3). The top ten up-regulated TF encoding genes were: Solyc06g074170.2.1 (*NAC060*, 6 h (>31 fold) and 48 h (>91 fold)), solyc12g088130.1.1 (AT5G10570.1, 48 h, (76 fold), solyc09g008830.2.1 (*HRA1*, 6 h (8 fold) and 48 h (>30. fold)), solyc06g084070.2.1 (*IAA1*, 6 h (>11 fold) and 48 h (ca. 30 fold)), solyc06g068940.2.1 (AT3G17980.1, 48 h (>26)), solyc00g050430.2.1 (*bHLH093*, 6 h (>16 fold) and 48 h (>22 fold)), solyc03g118190.2.1 (*RAP2.6* *L*, 48 h (ca. 20 fold)), solyc08g006470.2.1 (*REIL1*, 48 h, (>17fold)), solyc02g091980.1.1 (*MYB84*, 48 h, (>15 fold)), solyc06g054620.2.1 (*ZFN1*, 6 h (>15 fold) and 48 h (>15 fold)). We suggest that these are good candidate genes for further investigations regarding hypoxia tolerance in tomato and other crops.

In the following section, we discuss the most promising TF candidates among the top 10 highly up-regulated TFs in our study: *NAC060*, *RAP2.6* *L* and *MYB84*.

*NAC060*, has been shown to be induced by the sugar-ABA signalling pathway. However, NAC060 availability in the nucleus hinders sugar-ABA signalling leading to seedlings’ insensitivity to sugars^65^. A former study in rice showed that the link between low oxygen signals and the sugar-sensing cascade (via SnRK1) is important for regulation of sugar and energy production and therefore rice growth under flood conditions^66^. These data indicate that NAC060 is a promising candidate for further investigation with respect to its role in sugar sensing and hypoxia tolerance in tomato.

Induction of *RAP2.6* *L* in response to waterlogging and ABA has already been reported in *Arabidopsis*^67^. Overexpression of *RAP2.6* *L* resulted in a reduction of water loss as well as membrane leakage. Moreover, overexpression lines showed higher expression levels of genes encoding several antioxidant enzymes, the ABA biosynthesis gene and one of the hypoxia marker genes, *ADH1*. The authors concluded that *RAP2.6* *L* overexpression can delay premature senescence under waterlogging stress mainly through stomatal closure. Moreover, RAP2.6 L function might be linked to the ABI1-mediated ABA signalling pathway. Further studies are required to confirm the role of RAP2.6 L in tomato roots under hypoxia^67^.

Different stresses such as drought and salt stress as well as H_2_O_2_ and ABA have been shown to induce *MYB84* expression in soybean (*Glycine max*). *MYB84* has been shown to be involved in drought tolerance in soybean and its overexpression lines showed a higher resistance to drought including enhanced survival rate, longer root length, elevated proline and ROS level, higher activity of antioxidant enzymes and rehydration rate as well as reduced malondialdehyde (MDA) content. Moreover, GmMYB84 was shown to bind directly to the *GmRBOHB-1* and *GmRBOHB-2* promoters, suggesting a possible link between GmMYB84, H_2_O_2_ and antioxidant enzymes in controlling root growth in response to drought stress as well as under control conditions^68^. Feng *et al*., (2004), reported that *MYB84* is the closest homologue of *MYB68* and shows an overlapping expression pattern in root pericycle cells which suggests a redundant role in root development^69^. The role of *MYB84* in hypoxia resistance has not been previously studied. However, since *RBOHB* and *MYB84* showed up-regulation in our study, a similar model could be proposed for tomato roots in response to hypoxic stress. However, more studies are required to confirm the link between *MYB84* and *RBOHB* in response to hypoxia. *MYB84* appears to be a potential target for improving multi-stress resistance in plants.

Group VII members of the AP2/ERF (apetala2/ethylene response factor) transcriptions of *Arabidopsis thaliana* and rice have been shown to be involved in low oxygen sensing via the N-degron pathway^41,70^, such that they are stabilized under low oxygen conditions and induce the expression of hypoxia core genes. RAP2.12, RAP2.2 and RAP2.3 have been shown to accumulate in response to hypoxia; however, their accumulation has also been observed under normoxia^71,72^. On the other hand, two other ERF-VIIs members, HRE1 and HRE2, are hypoxia inducible^73^. *RAP2.2* and *RAP2.3* are *RAP2.12* homologues and have a redundant function in response to various stresses^72^. *RAP2.2*, a member of the same subfamily as the rice (*Oryza sativa*) submergence tolerance gene, *SUB1A*, has been shown to be involved in ethylene signal transduction as well as hypoxic stress. Overexpression of *RAP2.2* in *Arabidopsis thaliana* resulted in better hypoxia tolerance while its knockout lines showed an impaired resistance to hypoxia compared to wild type. Therefore, it has been concluded that RAP2.2 plays an important role in plant hypoxia resistance^71^. In the current study, *RAP2.2* showed>7 and>8 fold up-regulation after 6 h and 48 h hypoxic stress, respectively. Up-regulation of *RAP2.2* has also been shown to be involved in activation of ethylene biosynthesis genes such as *ACS7* and *ACO1* which resulted in ethylene production and regulation of genes responsive to waterlogging in *Arabidopsis*^70,71^. In accordance with this, we observed up-regulation of ACS*9* and *ACO1* after 48 h hypoxia. However, ethylene accumulation and its role during hypoxia in tomato root need further investigation.

We noted that, the *RAP2.3* gene showed down-regulation (>3 fold) at both time points in our study. Moreover, a member of the AP2/EREBP family, *Hypoxia Response Attenuator 1* (*HRA1*), was the third highest up-regulated TF gene, at both 6 h (>18 fold) and 48 h (>30 fold) hypoxia. *HRA1* has been reported to be highly up-regulated in response to hypoxia and to restrain the induction of core hypoxia-responsive genes in *Arabidopsis thaliana*. Since the constitutive accumulation of *RAP2.12* hinders a submergence and hypoxia tolerance response, it is supposed that a constant induction of stress response genes has a negative effect on stress response. *HRA1* has a negative regulatory role (negative feedback mechanism) on *RAP2.12* level and activity resulting in higher flexibility of hypoxia response under fluctuating O_2_ concentration^74,75^.

We compared differentially regulated genes (FDR <0.05) in response to 6 h hypoxia in our study with data presented in a very recent study based on the roots of submerged tomato seedlings^18^. We observed that ca. 12% (55 out of 450) of differentially regulated genes (FDR <0.05) in response to 6 h hypoxia in our study were also differentially regulated in the roots of submerged tomato seedlings **(**Supplementary Table 5**)**. The relatively low overlap between regulated genes in these two studies is possibly due to: 1) the differences in the root samples which were analysed (whole root in this study vs. 1 cm of the root tip). Since root tips are exposed to more hypoxia developmentally, it is possible that their response to transient hypoxia differs from the upper parts of the root. 2) Plant age and developmental phase can affect the root response to hypoxia. We used 5 week old plants in our study while the above-mentioned study was conducted at the seedling stage. 3) Technical differences in hypoxia application can partially be responsible for the observed discrepancy. In the current study, a hydroponic system was used and therefore only roots were exposed to low oxygen while in the other study the whole seedlings were submerged.

Our data suggest that hypoxia tolerance in tomato roots is dependent on a precise modulation of transcription via members of various TF families, apart from members of AP2/EREBP, to maintain metabolic balance within the cell. Highly regulated TF genes have the potential to be considered as early and/or late biomarkers of hypoxia response in tomato root and possibly other crop plants. We propose a model representing cellular processes potentially involved in hypoxia tolerance with an emphasize on the genes identified in this study **(**Fig. 10).

## Conclusion

We found evidence that the tomato root is sensitive to the duration of hypoxia which is reflected in the strong difference between the number and the function of the regulated genes after 6 h and 48 h hypoxia. Short-term hypoxia (6 h) resulted in molecular changes which in part were transient, implying on acclimation to the stress. However, long-term hypoxia (48 h) led to a stronger reprogramming of the transcriptome which, to a large extent, was not observed under short-term hypoxia. The long-term hypoxia tolerance mechanism of the tomato root seems to be an escape mechanism to avoid low oxygen concentration through aerenchyma and adventitious root formation. This is associated with down-regulation of antioxidant related genes, such as *CAT* and *MT2B*, and up-regulation of ethylene biosynthesis genes (*ACS9* and *ACO1*) as well as up-regulation of *RBOHB* which is involved in ROS production.

We identified transcription factor genes belonging to different families that were specifically regulated under short- or long-term hypoxia and some which were regulated during both time points. We hypothesize that some of these genes may have specific functions in regulating the target genes involved in hypoxia tolerance and may contribute to the differences between sensitive and tolerant varieties. Further studies are required to address this question and investigate the target genes for improving the hypoxia tolerance and selection of tomato cultivars with stronger adaptive responses to stress conditions.

In summary, our data suggest a transcriptional acclimation to short term hypoxia. However, hypoxia progression results in a transcriptional reprogramming to support an escape mechanism probably through aerenchyma and adventitious root formation. This indicates the ability of a cultivated crop such as tomato to temporally adjust its response mechanism to hypoxia, both metabolically and anatomically. However, further investigations are required to confirm the precise mechanism of the escape strategy in tomato root.

## Methods

### Plant material and growth conditions

Tomato plants (*Solanum lycopersicum* L. *cv*. Moneymaker) were cultivated on sand in the greenhouse at 500 μmol photons/m2/s and 25 °C under a 14/10‐h light/dark regime. All plants were treated with modified Hoagland nutrient solution containing 5 mM nitrate (NO_3_^−^) as described previously^76^. Three week old plants were transferred to hydroponic conditions such that the roots were submerged in plastic pots containing ~6 L of nutrient solution and aerated by mild bubbling using aquarist air pumps (Hailea ACO-9620, Raoping, Guangdong, China) and air outlets (Tetratec, Osnabrück, Germany). The hypoxia treatment was conducted on five weeks old plant roots using N_2_ gas (≥ 99.99 Vol. %) (Air Liquide, Germany). We harvested the roots after 6 h and 48 h of root exposure to N_2_ gas. Oxygen concentration was measured using CellOx 325 DO electrodes (WTW, Germany) following manufacturer’s instructions. For each root sample (after 6 h or 48 h hypoxia) three biological replicates, each containing a pool of three technical replicates, were harvested and immediately frozen in liquid nitrogen and stored in −80 °C **(**Fig. 10). We harvested the roots 6 h and 48 h after the initiation of root exposure to N2 gas. Hypoxia treatment was initiated at 8:00 a.m. The 6 h hypoxia and control samples were collected at 2:00 p.m. 48 h hypoxia and control samples were collected two days later at 8.00 a.m. The hypoxia and control samples at each time point were harvested and compared separately to remove the impact of other unknown or known additive factors between time points such as circadian rhythm^1^.

Relative chlorophyll levels of leaf #3 (the third leaf above the cotyledon) were determined using a Konica Minolta SPAD-502 chlorophyll meter. For each sample, SPAD values from three similar positions on the leaf #3 were recorded and their averages were calculated.

### RNA isolation and cDNA synthesis

For RNA-Sequencing (RNA-Seq) and qRT-PCR analysis, total RNA was extracted from 250 mg frozen root tissue using a phenol-chloroform extraction method^77^. RNA concentration was quantified photometrically using a NanoDrop (ND-1000, Thermo Scientific, Wilmington, DE, USA) and RNA integrity was tested on 1.2% agarose gel. 2 μg DNaseI-digested total RNA was used for cDNA synthesis using oligo-(dT)_18_ and the RevertAid H Minus First Strand kit (Thermo Scientific, Waltham, USA).

### Read mapping and identification of differentially expressed genes

Adaptor clipped reads obtained from the NextSeq. 500 Illumina platform (LGC Biosearch Technologies, Berlin, Germany), were processed to omit short fragments (final length <20 bases) and low quality reads. The filtering of rRNA sequences was conducted using RiboPicker 0.4.3. The remaining reads were mapped onto the tomato reference genome (ITAG 2.4) (The Tomato Genome Consortium, 2012) using CLC Genomics Workbench (Qiagen, V. 7.5.5). Quantification of the gene expression levels were conducted using the CLC Genomics Workbench (Qiagen, V. 7.5.5). Sequencing data are deposited in the Sequence Read Archive (SRA) database (bioproject accession PRJNA553994) at the National Centre for Biotechnology Information (NCBI). The bioproject’s metadata are available at https://dataview.ncbi.nlm.nih.gov/object/PRJNA553994?reviewer=684sto9a948tin240f0tlt1o1h.

For normalization and estimation of P-values the TMM (trimmed means of M values^78^) and the edger algorithm^79^ were used respectively. The log fold change values are provided as calculated by the edgeR algorithm. The P-values were adjusted for multiple testing^80^. All calculations were performed with the CLC Genomics Workbench software (Qiagen, V. 7.5.5). Differentially expressed genes (DEGs) with ≥2-fold expression change and P_adj_ <0.05 **(**Supplementary Table 6**)** were selected for subsequent analysis. The FDR threshold was used for the P-value in multiple tests (P_adj_). GO term enrichment analysis was performed using the Panther database^81,82^ to identify significantly enriched GO terms (P_adj_<0.05) associated with DEGs in response to 6 h and 48 h hypoxia. For biological pathway analysis of differentially regulated genes, MapMan categories based on ITAG 2.3 annotations^29^ were used.

### qPCR primer design and assay

Primers for quantitative real-time PCR (qPCR) were designed using QuantPrime^83^ **(**Supplementary Table 7**)**. qPCR reactions were performed in 5 μl total reaction volumes including 2.5 μl Power SYBR Green Master Mix (ThermoFisher Scientific), 0.5 μM forward and reverse primers and 0.5 μl cDNA. *ACTIN* was used as reference gene^84^. The thermal profile used for all qPCRs was: 2 min 50 °C; 10 min 95 °C; (15 s 95 °C; 1 min 60 °C)_40×_. Data were analysed by the 2^−ΔΔCt^ method^85^.

## Supplementary information

Supplementary Information.

## Data Availability

All materials and data sets represented in the current study are available in the main text or the supplementary materials. RNA-Seq data are deposited in the Sequence Read Archive (SRA) database (bioproject accession PRJNA553994) at the National Centre for Biotechnology Information (NCBI). The bioproject’s metadata are available at https://dataview.ncbi.nlm.nih.gov/object/PRJNA553994?reviewer=684sto9a948tin240f0tlt1o1h.
