# Author Correction: RNA-Seq reveals novel genes and pathways associated with hypoxia duration and tolerance in tomato root

**DOI:** 10.1038/s41598-020-75106-5

**Published:** 2020-11-20

**Authors:** Vajiheh Safavi-Rizi, Marco Herde, Christine Stöhr

**Affiliations:** 1 Department of Plant physiology, Institute of Botany and Landscape Ecology, University of Greifswald, Greifswald, Soldmannstrasse 15, D-17487 Greifswald, Germany; 2 Department of Molecular Nutrition and Biochemistry of Plants, Institute of Plant Nutrition, Leibniz University Hannover, Herrenhäuser Strasse 2, 30419 Hannover, Germany

Correction to: *Scientific Reports* 10.1038/s41598-020-57884-0, published online 03 February 2020

The original version of this Article contained extensive errors in the reference list, which was incorrectly given in alphabetical order.

In addition, this Article contained multiple errors in the order of figures referred to in the Results and Discussion sections.

In the Results section under the subheading “Hypoxia affects the link between N metabolism and NO formation/scavenging.”,

“Altogether, these results suggest that N metabolism might be involved in NO production and scavenging in tomato root adaption to hypoxic stress with a gene regulation specific response to the duration of hypoxia (Figs. 5 and 6).”

now reads:

“Altogether, these results suggest that N metabolism might be involved in NO production and scavenging in tomato root adaption to hypoxic stress with a gene regulation specific response to the duration of hypoxia (Fig. 5).”

Furthermore, every instance of “Fig. 7”, “Fig. 8”, “Fig. 9” or “Fig. 10” in the Results and Discussion sections now reads “Fig. 6”, “Fig. 7”, “Fig. 8” or “Fig. 9”, respectively.

These errors have now been corrected in the HTML and PDF versions of the Article.

